# Do pathogen effectors play peek-a-boo?

**DOI:** 10.3389/fpls.2014.00731

**Published:** 2014-12-18

**Authors:** Guus Bakkeren, Barbara Valent

**Affiliations:** ^1^Pacific Agri-Food Research Centre, Agriculture and Agri-Food CanadaSummerland, BC, Canada; ^2^Department of Plant Pathology, Kansas State UniversityManhattan KS, USA

**Keywords:** avirulence gene, oomycete and fungal diseases, methylation, host resistance, phytophthora, effectors, epigenetic regulation, phytopathology

Epigenetics is a burgeoning field of research and many organisms are found to regulate genes in a transient (“above genetics”) way due to (temporarily) decorating DNA or proteins that associate with DNA. This can change transcription of affected genes and since such DNA modifications are reversible, the expression potential over time and generations can therefore be extremely variable. This allows many organisms to readily adapt and experiment with changes to the environment they are living in or challenges they are facing, such as pathogens (Iwasaki and Paszkowski, 2014). This research topic deals mainly with epigenetic mechanisms of plant responses but in the field of plant pathology, recent experimental data illustrate the involvement of epigenetic changes in both plant host and their pathogens (e.g., Luna et al., 2012; Qutob et al., 2013; Yu et al., 2013; Soyer et al., 2014). Indeed, pathogen pressure is very much an environmental cue that will induce epigenetic changes in plant hosts, but, in response, pathogens seem equipped to respond in kind.

The interaction between many pathogens and their hosts is modulated by large suites of effectors, often small proteins that are secreted by pathogens and end up in host tissues, in the apoplast or cytoplasm. They play various roles supporting the infection and the pathogen's propagation, such as suppressing host defense responses (Giraldo and Valent, 2013). Naturally, hosts have evolved mechanisms that use such effectors or their actions as cues to mount a counter defense to stop the pathogen in its track. Such cues are often perceived by resistance gene products, the sentries that are part of the host surveillance system and whose activation triggers the defense responses. In a tit-for-tat, the pathogen evolves to prevent the offending effector from being expressed and this can be done in a variety of ways: mutating its recognition, deletion from the genome, or preventing its expression. Especially the latter option is attractive since the coding information would still be available and the effector could potentially be “recycled” later on if the pressure is relieved, that is, if the host with the resistance gene that recognized the offending effector activity is gone from the environment.

In the very timely perspective by Gijzen et al. (2014), the authors elaborate on the last scenario and work from their own laboratory gives an example of how an offending effector becomes silenced in a pathogen as to regain virulence and that this effect persists over many generations (Qutob et al., 2013). Interestingly, many effectors have been found embedded in regions with large numbers of transposable elements. Transposable elements have been dubbed “genome modifiers” and have been shown to affect gene expression while themselves being very prone to epigenetic control (Fedoroff, 2012). Intriguing findings indeed suggest the possible control of the expression of fungal effector genes embedded in epigenetically-controlled transposable element-rich chromatin (Soyer et al., 2014). The perspective Gijzen et al. highlights several recent publications that point to possible mechanisms by which such transient changes in effector expression may occur. It offers an explanation for several older literature reports in which such variable and apparently reversible virulences (avirulences) were observed both in the laboratory and in the field. In addition, and as a warning, this perspective discusses the consequences of these findings for the current development of diagnostic assays based solely on simple DNA analysis and the error rates this would produce; the idea for the need for more-sophisticated assays is floated. Finally, the question is posed whether this phenomenon is more wide-spread than currently appreciated, a prospect that makes the re-assessment of such diagnostic assays even more dire.

## Conflict of interest statement

The Guest Associate Editor Yuhai Cui declares that, despite being affiliated to the same institution as the author Guus Bakkeren, the review process was handled objectively and no conflict of interest exists. The authors declare that the research was conducted in the absence of any commercial or financial relationships that could be construed as a potential conflict of interest.
